# Diagnose This quiz

**Published:** 2013

A patient presents with a dilated pupil, depicted in the figure; 45 minutes after instillation of 1% pilocarpine, it remains unchanged. What is the most likely diagnosis?

**Figure:**
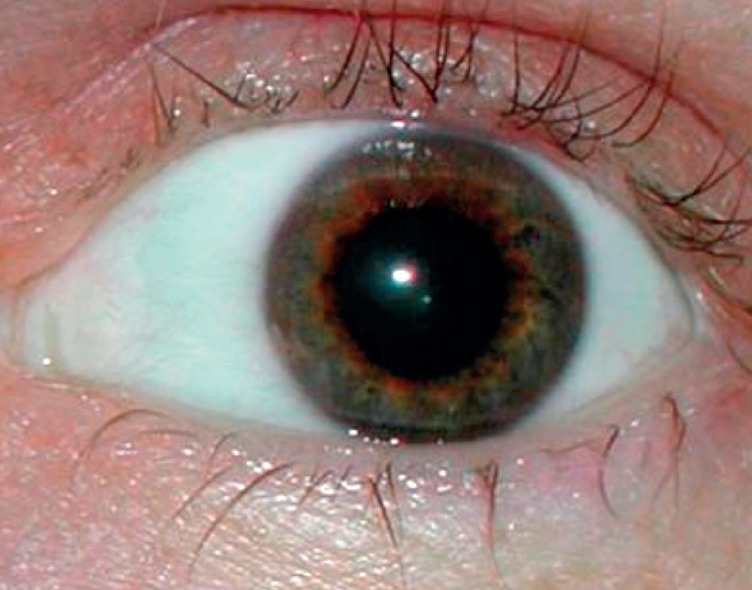

□ Tonic (Adie) pupil
□ Pharmacologic dilation
□ Horner syndrome
□ Third cranial nerve palsy

## ANSWER

### Pharmacologic dilation

The figure depicts a dilated pupil that was unaffected by 1% pilocarpine. The 1% pilocarpine test distinguishes all causes of pathologic pupillary dilation from pharmacologic dilation. Generally a pharmacologically dilated pupil will not constrict to 1% pilocarpine, whereas tonic pupils, third nerve palsy pupils, and Horner syndrome pupils will constrict. Horner syndrome also causes miosis in the affected eye. With dilute pilocarpine (e.g., 0.1%), a tonic pupil will demonstrate denervation supersensitivity and constriction.

**Figure:**
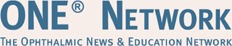


Reproduced by kind permission of the Ophthalmic News and Education (ONE®) Network of the American Academy of Ophthalmology. Visit www.aao.org/one

